# Downregulation of DACH2 Expression in an Adrenocortical Cell Model of PCOS with Adrenal Hyperandrogenism, and in Human Granulosa Cells from Patients with Hyperandrogenic-PCOS: a Link Between Ovaries and Adrenal Glands in PCOS

**DOI:** 10.1007/s43032-026-02078-8

**Published:** 2026-03-16

**Authors:** Caglar Berkel

**Affiliations:** Department of Molecular Biology and Genetics, Tokat Gaziosmanpasa University, Tokat, Türkiye

In vitro PCOS (polycystic ovary syndrome) cell models, such as H295R cells (a testosterone-producing adrenocortical cell line), are important to study molecular mechanisms in this endocrine and metabolic disorder in the menstruating population [34]. H295R cells serve as a robust cell model for androgen overproduction and human steroidogenesis, in particular to model PCOS with adrenal hyperandrogenism, which is observed in around 20–30% of the affected women [3, 5, 8, 17, 24–26, 34]. Increased levels of serum androgens in patients with PCOS have been shown to originate from both the ovaries and the adrenal glands, from a common precursor, cholesterol [6, 20]. In other words, adrenal glands are an additional source of hyperandrogenemia in a significant proportion of women with PCOS, although ovaries are the primary source of increased androgens in PCOS [10, 24, 27, 28, 33]. Adrenal androgen (AA) excess in patients with PCOS is detectable primarily by increased levels of dehydroepiandrosterone sulfate (DHEAS) [14, 33]. Some studies even suggest that abnormally high production of androgens by the adrenals at earlier life stages (in the fetus or prepubertal child) may be a precursor of the PCOS phenotype later in adolescence and young adulthood [9, 21]. The complete understanding of the molecular mechanisms of adrenal hyperandrogenism in PCOS is still lacking. Besides, genes whose expression are regulated similarly in response to androgens in adrenocortical cells (of adrenal glands) and granulosa cells (of ovaries) in PCOS remain to be studied.

Here, by using two very recently published independent RNA-seq datasets, we found that DACH2 expression is downregulated in H295R cells in response to forskolin (in vitro), and in human ovarian granulosa cells in response to high androgen (in vivo) (Fig. 1). In the construction of the one of these datasets, H295R cells were treated with forskolin (10 μM) for 72 h, and differently expressed genes (DEGs) in response to forskolin treatment in H295R cells were identified (compared to DMSO-treated control H295R cells) [22]. Forskolin, by mimicing the action of luteinizing hormone (LH), positively impacts testosterone production in these cells, creating an in vitro cell model of PCOS with adrenal hyperandrogenism [1, 11, 22, 30]. In the other dataset, the expression of genes in granulosa cells from women with hyperandrogenic-PCOS (HA-PCOS; serum total testosterone (TT) levels higher than or equal to 0.69 ng/mL) was compared to those in granulosa cells from non-PCOS women [29]. Similarly, differentially expressed genes between granulosa cells from women with normoandrogenic PCOS (NA-PCOS; serum total testosterone (TT) levels lower than 0.69 ng/mL) and granulosa cells from non-PCOS women were identified in this dataset [29]. We then filtered out differently expressed genes only present in “HA-PCOS vs control” comparison, but not in “NA-PCOS vs control” comparison, in order to determine genes whose expression were specifically regulated in response to high androgen levels (serum TT ≥ 0.69 ng/mL).

**Fig. 1:**
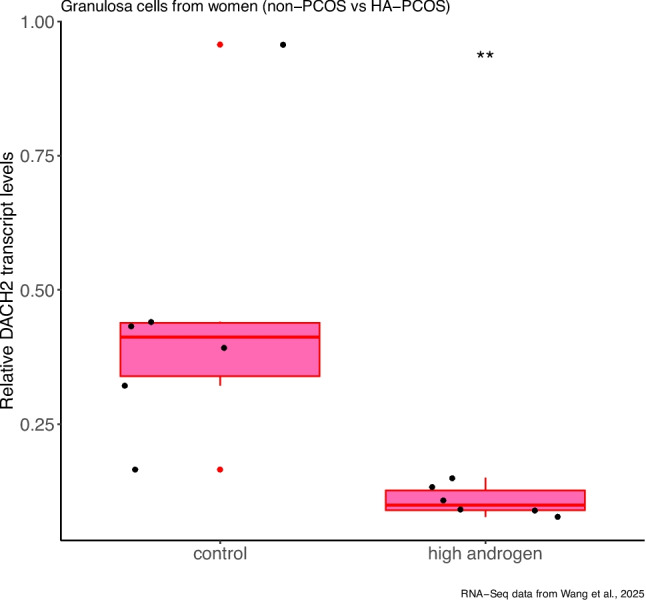
Relative DACH2 mRNA levels between ovarian granulosa cells from non-PCOS women and women with hyperandrogenic-PCOS (HA-PCOS; those having serum total testosterone levels higher than or equal to 0.69 ng/mL ). Data from [29]

Later, we identified genes whose expression change similarly in H295R cells in response to forskolin treatment, and in human granulosa cells in response to high androgen (HA). We found that there is only one such gene when we intersect differentially expressed genes from both datasets: DACH2 (Dachshund Family Transcription Factor 2) (Fig. 1). Its expression were shown to decrease significantly in H295R cell line and human granulosa cells from women in response to forskolin and high androgen, respectively (log2FoldChange values of -2.30 and -2.05, and adjusted p values of 2.83e-57 and 0.000420, respectively). This shows that DACH2 expression might be downregulated in response to high androgen levels similarly in adrenocortical cells and granulosa cells in the pathogenesis of PCOS. Downregulated DACH2 expression might be a link between adrenal hyperandrogenism and follicular hyperandrogenism in PCOS, or might represent a shared mechanism in adrenocortical cells and ovarian granulosa cells in response to high androgen in PCOS. This high androgen-induced decreases in DACH2 expression in both adrenocortical cells and granulosa cells should be better characterized, and its potential pathological consequences in both tissues need to be mechanistically determined in the context of PCOS.

Certain SNPs were identified in DACH2 gene as potential risk factors in patients with POI (premature ovarian insufficiency), a disease with certain similarities to PCOS [2, 4, 12, 18]. These studies suggested that particular variants of DACH2 gene might be able to modify the normal differentiation of ovarian follicle, thus negatively influencing fertility [4, 12, 15, 19, 23, 31]. Mutations in DACH2 gene were also found to be associated with poor female reproductive tract development in other studies [7, 13, 16, 32]. It was also identified as an independent factor of poor prognosis in serous ovarian cancer [16]. However, whether the downregulation of DACH2 in response to high androgen in both adrenocortical cells and granulosa cells might contribute to the pathogenesis of PCOS remains to be studied. Further mechanistic studies on the function of DACH2 in these two cell types are required in in vitro and in vivo models of PCOS.

## Data Availability

The data used in the present study is publicly available as detailed.

## Code Availability

Code is available from the author upon request.
